# A new species of the genus *Baeoura* from Morocco, with a key to the West Palaearctic species (Diptera, Tipuloidea, Limoniidae)

**DOI:** 10.3897/zookeys.532.5994

**Published:** 2015-11-05

**Authors:** Ouafaa Driauach, Boutaïna Belqat

**Affiliations:** 1 Laboratory “Ecology, Biodiversity and Environement”, Department of Biology, Faculty of Sciences, University Abdelmalek Essaâdi, Tétouan, Morocco

**Keywords:** Diptera, Limoniidae, *Baeoura*, new species, Morocco

## Abstract

The genus *Baeoura* is represented in Morocco by two species, *Baeoura ebenina* Starý, 1981, and *Baeoura staryi* **sp. n.** The new species is described and illustrated, and a key to the West Palaearctic species of *Baeoura* is presented.

## Introduction

The genus *Baeoura* Alexander, 1924 belongs to the subfamily Chioneinae. It originally was erected by Alexander (1924) as a subgenus of *Erioptera* Meigen, 1803, with the type species *Erioptera nigrolatera* described by Alexander, 1920. It later was transferred to the genus *Cryptolabis* Osten-Sacken, 1859 (Starý 1981). The genus comprises small species with antennae of 15 articles, a long Rs, the male hypopygium with a single gonostylus, a reduced ovipositor, and eggs which are large and blackened with a smooth surface. The immature stages are aquatic (Mendl and Tjeder 1974).

Worldwide, 70 species of the genus *Baeoura* have been reported, including 43 in the Oriental Region, ten in the Afrotropical Region, four in Australia, four in the East Palaearctic, eight in the West Palaearctic, and a single species in Chile (Oosterbroek 2014). In North Africa, the genus was recorded by Krzeminski and Starý (1984) who described a new species, *Baeoura szadziewskii*, from northeastern Algeria. Two species belonging to the genus *Baeoura* have been collected in northwestern Morocco: *Baeoura ebenina* Starý, 1981, a new record for North Africa, and *Baeoura staryi* sp. n. Thus, in Morocco, the genus *Baeoura* is represented by two species. Here, *Baeoura staryi* sp. n. is described and a key to the West Palaearctic species of *Baeoura* (mostly males) is provided.

## Material and methods

The specimens were collected using an entomological hand net. Genital preparations were made by macerating the apical portion of the abdomen in cold 10% KOH for 12–15 h. After examination, the genitalia were transferred to fresh glycerin and stored in a microvial. The holotype (male in alcohol) is deposited in the collections of the laboratory at Ecology, Biodiversity and Environment, Faculty of Science, University Abdelmalek Essaâdi, Tétouan, Morocco. A paratype male (dry) is deposited in the collection of J. Starý, Olomouc, Czech Republic.

Terminology of morphological features generally follows that of McAlpine (1981).

## Systematic

### Key to West Palaearctic species of *Baeoura* (mostly males)

**Table:** 

1	Body yellowish brown to brown	**2**
–	Body brownish black to black	**6**
2	Tergite 9 narrowed distally, posterior margin with comparatively narrow median emargination and short truncate lobe on each side; ventral and dorsal lobes of gonocoxite well developed, ventral lobe longer than body of gonocoxite (Fig. 6)	**3**
–	Tergite 9 relatively broad, posterior margin with broad median emargination and projecting corner on each side; ventral lobe of gonocoxite shorter than body of gonocoxite, dorsal lobe indistinct	**5**
3	Gonostylus curved, with broad spatulate apex bearing small sharp tooth, directed backwards; for male and female terminalia, see Mendl and Tjeder (1976), Figs 1–9. Bulgaria, Greece, Romania, Serbia	***Baeoura malickyi* Mendl & Tjeder**
–	Gonostylus more or less S-shaped, pointed at apex	**4**
4	Sc_1_ ending beyond fork of Rs; gonostylus slender, gradually narrowed to pointed apex; for male terminalia, see Krzemiński and Starý (1984), Figs 1–4. Algeria	***Baeoura szadziewskii* Krzemiński & Starý**
–	Sc_1_ ending just before fork of Rs; gonostylus long, slender, with bulge-shaped extension at about mid-length provided with group of setulae on outer surface, tapered to slender distal half, strongly bent posteriorly, and almost filiform before pointed apex (Fig. 1–9). Morocco	***Baeoura staryi* sp. n.**
5.	Gonostylus narrowed just before tip, obtuse at apex; aedeagus dilated in proximal half, spindle-shaped in dorsal aspect, produced into long filament; for male and female terminalia, see Mendl and Tjeder (1974), Figs 2–10. Greece (Crete), Turkey	***Baeoura alexanderi* Mendl & Tjeder**
–	Gonostylus narrowed in distal third, beak-shaped, obtuse at apex; aedeagus simple, slender; for male and female terminalia, see Mendl (1986), Figs 1–7. Turkey	***Baeoura schachti* Mendl**
6.	Gonostylus with conspicuous, roughly triangular extension at outer base; aedeagus filiform; for male terminalia, see Mendl (1986), Figs 8–10. Spain	***Baeoura longefiligera* Mendl**
–	Gonostylus and aedeagus of different shape	**7**
7	Sc_1_ ending beyond fork of Rs; proximal section of M_3+4_ (before m-cu) subequal in length to or longer than m-cu	**8**
–	Sc_1_ ending before fork of Rs; proximal section of M_3+4_ (before m-cu) about half length of m-cu; for male and female terminalia, see Starý (1981), Figs 6–10. Portugal, Spain	***Baeoura ebenina* Starý**
8	Tergite 9 with short, broad lobe on each side of its posterior margin; for male and female terminalia, see Starý (1981), Figs 1–5. France (Corsica), Italy (Sicily)	***Baeoura directa* (Kuntze)**
–	Tergite 9 with slender projection on each side of its posterior margin; for male and female terminalia, see Mendl (1985), Figs 1–6. Greece (Crete)	***Baeoura armata* Mendl**

**Figures 1–5.**
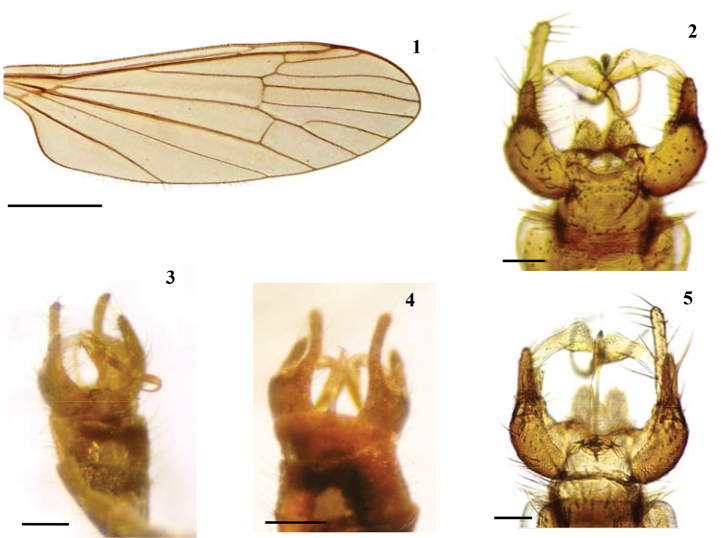
*Baeoura staryi* sp. n. **1** Wing **2, 3** Male terminalia, dorsal view **4, 5** Male terminalia, ventral view. Scale bars: 1 mm (**1**); 0.1 mm (**2–5**); 0.2 mm (**3–4**).

**Figures 6–9.**
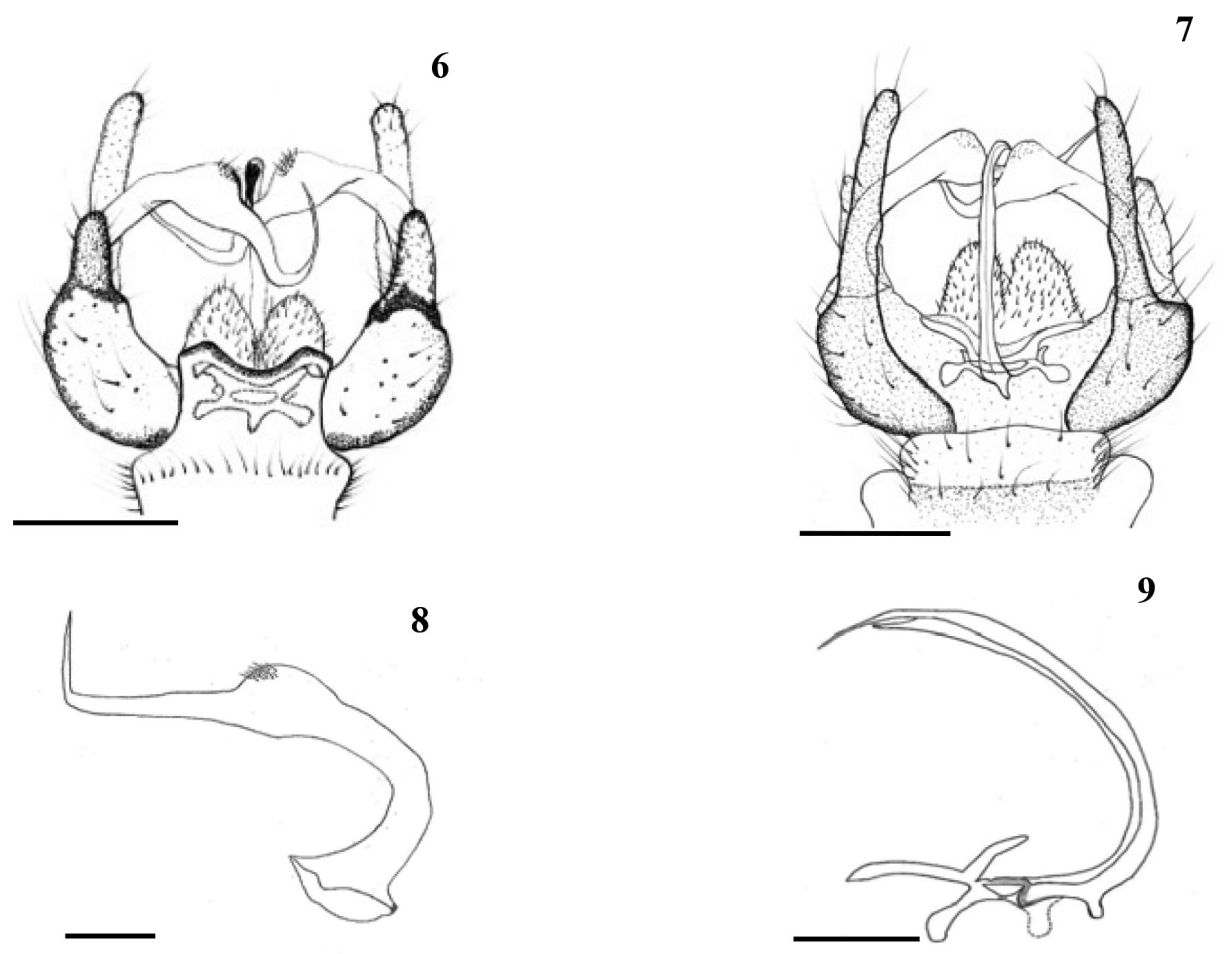
*Baeoura staryi* sp. n. (holotype male) **6** Male terminalia, dorsal view **7** Male terminalia, ventral view **8** Gonostylus, dorsal view **9** Aedeagus, lateral view. Scale bars: 0.2 mm (**6–7**); 0.1 mm (**8–9**).

**Figure 10.**
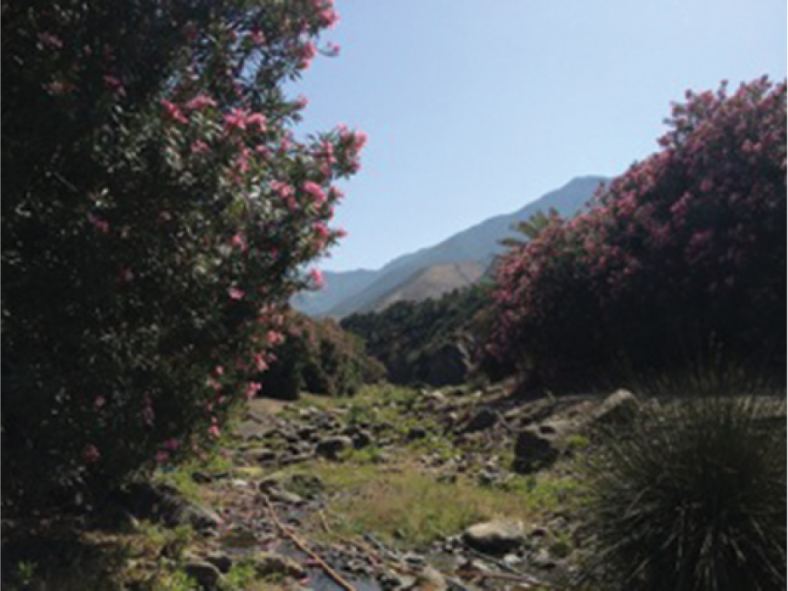
Oued Jnane Niche, type locality of *Baeoura staryi* sp. n.

### Baeoura ebenina

Taxon classificationAnimaliaDipteraLimoniidae

Starý, 1981

#### Material examined.

Rif Mts, Chefchaouen Province, Mezine village: 1♂, 2♀♀, Tributary Oued Tazarine, 35°05.670'N/5°21.991'W, 731 m, 11.vi.2013, middle course of the river; 1♂, 1♀, Daya near Aïn Afersiw, 35°06.069'N/5°20.337'W, 716 m, 11.vi.2013, pond. O. Driauach and B. Belqat leg.

#### Distribution.

*Baeoura ebenina* was previously known only from Spain and Portugal (Oosterbroek 2014). We provide the first record for Morocco and North Africa.

#### Ecology.

According to Starý (2014), this species is collected near rivers and brooks. In Morocco, we collected adults by sweeping the vegetation around lotic and lentic habitats. One habitat was in the middle of a temporary river with a substrate of stones, gravel, and sand. The wet section was reduced to a thin layer of water, due to the beginning of the dry season and to water being pumped out by countrymen; the current velocity was slow to medium. There was a proliferation of filamentous green algae. Riparian vegetation consisted primarily of *Nerium oleander* and herbaceous vegetation. A second habitat was a pond surrounded by conifer reforestation, with the edges overgrown by grasses and herbaceous vegetation.

### Baeoura staryi sp. n.

Taxon classificationAnimaliaDipteraLimoniidae

http://zoobank.org/D79FC3AE-DF30-4796-A119-B6902D619945

Figs 1–5 , 6–9

#### Diagnosis.

Body dark brown, patterned with yellow. Tergite 9 narrow distally, with median emargination and short truncate lobe on each side. Gonocoxite with two lobes; dorsal lobe rather short and broad; ventral lobe long and slender. Single gonostylus long, slender, with bulge-shaped extension at about mid-length provided with group of setulae, and filiform before pointed apex.

#### Description.

Male: Body dark brown, patterned with yellow. Body length 3.5–3.8 mm, wing length 4.5–4.7 mm.

Head: Dark greyish brown; rostrum obscurely yellow; palpus yellowish brown, with terminal palpomere elongate. Antenna dark brown, with 15 articles, bent backwards, reaching to about anterior margin of thorax. Scape cylindrical; pedicel large, ovoid, much broader than scape. First flagellomere rather long-ovoid, smaller than scape but distinctly larger than other flagellomeres, these diminishing toward apex of antenna. Verticils on flagellomeres sparse and short, not reaching length of respective flagellomere.

Thorax: Pronotum brown dorsally, yellow laterally. Mesonotum dark brown with broad, pale yellow stripe on each side close above wing, from pronotum to scutellum. Scutum with distinct yellow marking near base of wing. Scutellum light yellow, light brown only medially on extreme anterior margin. Pleuron generally greyish brown, light yellow on dorsopleural membrane (part of lateral stripe).

Legs: Anterior coxa brown, middle coxa pale brown, hind coxa yellow. Trochanters yellowish brown. Femora yellowish brown, with darker distal half and blackish brown at distinctly enlarged apex. Tibiae yellowish brown. Tarsi dark brown. Tibiae longer than femora. Legs rather densely and darkly haired.

Wing (Fig. 1): Hyaline with faint yellowish-grey tinge; veins primarily brown. Sc ending just before fork of Rs. Halter rather stout, with white knob.

Abdomen: Dark brown dorsally and ventrally, paler laterally.

Male terminalia (Figs 2–7): Yellowish brown. Tergite 9 narrow distally, its posterior margin with median emargination, and with short, truncate lobe on each side of it. Gonocoxite with two lobes; dorsal lobe rather short and broad; ventral lobe long and slender, gently curved dorsally. Single gonostylus of peculiar shape, long, slender, with bulge-shaped extension at about mid-length provided with group of setulae at outer surface, then tapered into slender distal half, strongly bent posteriorly, and almost filiform before pointed apex (Fig. 8). Aedeagus long, slender, curved dorsally (Fig. 9).

Female: Unknown.

#### Specimens examined.

**Holotype.** Male in alcohol, from Morocco, Rif Mts, Chefchaouen Province, Jnane Niche village, toward Jebha, Oued Jnane Niche, 35°17.040'N / 4°51.479'W, 46 m above sea level, 19.iv.2013, O. Driauach and B. Belqat leg.

**Paratype.** Dry, one male, same locality as holotype, 14.vi.2013, O. Driauach and B. Belqat leg.

#### Etymology.

This species is named in honor of Dr. Jaroslav Starý (Olomouc, Czech Republic), with our thanks for his help in the identifications of the Moroccan Limoniidae.

#### Remarks.

*Baeoura staryi* sp. n. is distinctive in having the ventral lobe of the gonocoxite long and slender, the longest among the West Palaearctic species. In the shape of tergite 9, the new species resembles *Baeoura malickyi* Mendl & Tjeder, 1976, and *Baeoura szadziewskii* Krzemiński & Starý, 1984, but differs from these species especially by the peculiar shape of the gonostylus.

#### Distribution and ecology.

Morocco. The species was collected from vegetation by a river on dry, stony ground with small streams, at an altitude of 46 m (Fig. 10).

## Supplementary Material

XML Treatment for Baeoura ebenina

XML Treatment for Baeoura staryi

## References

[B1] Krzeminski W Stary J (1984) A new species of *Baeoura* Alexander, 1924 (Diptera, Limoniidae), from Algeria. Polskie Pismo Entomologiczne 54: 359–361.

[B2] McAlpine JF (1981) Morphology and terminology: Adults. In: McAlpine JF et al. (Eds) Manual of Nearctic Diptera 1. Biosystematic Research Institute, Ottawa, Ontario, Monograph 27: 9–63.

[B3] Mendl H Tjeder B (1974) A new species of *Baeoura* from the island of Crete (Diptera, Tipulidae). Entomologica Scandinavica 5: 247–250. doi: 10.1163/187631274X00281

[B4] Mendl H Tjeder B (1976) *Baeoura malickyi* n.sp. from the Greek mainland and the island of Rhodos (Diptera: Tipulidae). Entomologica Scandinavica 7: 237–238. doi: 10.1163/187631276X00397

[B5] Mendl H (1985) Eine neue *Baeoura*-Art aus dem Mittelmeergebiet (Diptera Nematocera: Limoniidae). Articulata 2: 196–198.

[B6] Mendl H (1986) Zwei neue *Baeoura*-Arten aus der Westpalaarktis (Diptera Nematocera: Limoniidae). Articulata 2: 293–298.

[B7] Oosterbroek P (2014) Catalogue of the Craneflies of the World, (Diptera, Tipuloidea: Pediciidae, Limoniidae, Cylindrotomidae, Tipulidae). http://ip30.eti.uva.nl/ccw/index.php [accessed 30 October, 2014]

[B8] Starý J (1981) *Baeoura directa* (Kuntze, 1914) with the description of a new species from Spain (Diptera, Limoniidae). Reichenbachia 19: 97–103.

[B9] Starý J (2014) Some records of Limoniidae and Pediciidae (Diptera) from Portugal and Spain. Acta Musei Silesiae, Scientiae Naturales 63: 83–95. doi: 10.2478/cszma-2014-0010

